# Common brain activations for painful and non-painful aversive stimuli

**DOI:** 10.1186/1471-2202-13-60

**Published:** 2012-06-07

**Authors:** Dave J Hayes, Georg Northoff

**Affiliations:** 1 Mind, Brain Imaging and Neuroethics Research Unit, Institute of Mental Health Research, University of Ottawa, 1145 Carling Avenue, Ottawa, K1Z 7K4, Canada

**Keywords:** Meta-analysis, Translational, Aversion, Pain, Neuroimaging, Animal models

## Abstract

**Background:**

Identification of potentially harmful stimuli is necessary for the well-being and self-preservation of all organisms. However, the neural substrates involved in the processing of aversive stimuli are not well understood. For instance, painful and non-painful aversive stimuli are largely thought to activate different neural networks. However, it is presently unclear whether there is a common aversion-related network of brain regions responsible for the basic processing of aversive stimuli. To help clarify this issue, this report used a cross-species translational approach in humans (i.e. meta-analysis) and rodents (i.e. systematic review of functional neuroanatomy).

**Results:**

Animal and human data combined to show a core aversion-related network, consisting of similar cortical (i.e. MCC, PCC, AI, DMPFC, RTG, SMA, VLOFC; see results section or abbreviation section for full names) and subcortical (i.e. Amyg, BNST, DS, Hab, Hipp/Parahipp, Hyp, NAc, NTS, PAG, PBN, raphe, septal nuclei, Thal, LC, midbrain) regions. In addition, a number of regions appeared to be more involved in pain-related (e.g. sensory cortex) or non-pain-related (e.g. amygdala) aversive processing.

**Conclusions:**

This investigation suggests that aversive processing, at the most basic level, relies on similar neural substrates, and that differential responses may be due, in part, to the recruitment of additional structures as well as the spatio-temporal dynamic activity of the network. This network perspective may provide a clearer understanding of why components of this circuit appear dysfunctional in some psychiatric and pain-related disorders.

## Background

### Aversion: painful and non-painful stimuli

Identification of potentially harmful stimuli is necessary for the well-being and self-preservation of all organisms. Organisms with relatively simple nervous systems (e.g. worms, fruit flies) display motivated approach and avoidance behaviours to rewarding and aversive stimuli, respectively, implying the existence of some evolutionarily conserved mechanisms [1,2]. Aversive stimuli are those which an organism will generally expend energy to minimize or avoid [3]; in this context, aversion is operationally opposite to reward [4]. However, the strength of aversive stimuli and the context in which they occur can produce a variety of psychophysical (e.g. negative emotion, pain) and behavioural (e.g. reduced behaviour following punishment, avoidance) responses. While recent work has suggested the existence of a common aversion-related network of brain regions responsible for the basic processing of aversive stimuli [5], this work focused only on studies involving non-painful stimuli and studies including painful stimuli were not considered; as such, it is unclear if those results extend to pain-related processing.

### Pain-associated brain activity

Pain, which typically results from activating the nociceptive system (but can also involve non-nociceptive mechanisms, such as in neuropathic pain), is experienced across mammals and is critical for survival [6]. Studies in humans and non-human animals have generally supported the notion that pain is processed differentially in the brain according to affective (e.g. amygdala, anterior insula, hippocampus) and sensory (e.g. somatosensory cortices, posterior insula) dimensions (e.g. [7-9]; though see also [10] for a review on the influential 3-dimension theory of pain). Nonetheless, the assumption that this network (sometimes referred to as the ‘Pain Matrix’) is specifically activated by painful stimuli has been questioned [11-13]. Using fMRI in humans, Mouraux et al. (2011) uncovered strong support for the notion that the typical regions of the Pain Matrix are largely involved in salience processing [12]. They showed that multimodal non-painful aversive stimuli and painful stimuli activate similar regions in the MCC, insula, thalamus, and sensory cortex and that the BOLD signals in these regions correlated largely with the perceived saliency of the stimulus (regardless of modality or stimulus type).

### Studies suggest shared regions for pain and non-pain aversion

While much work has identified pain as a uniquely important experience (e.g. [10,14]), numerous studies using non-painful aversive stimuli (e.g. unpleasant sounds, sights, etc.) have implicated many of the same cortical and subcortical regions [5], suggesting that the processing of various painful and non-painful aversive stimuli require many of the same neurobiological substrates. In this regard, human studies have been key to understanding the role of cortical regions (e.g. prefrontal and insular cortices; e.g. [15,16]). Alternately, studies in animals have highlighted the importance of subcortical areas such as the periaqueductal grey, hypothalamus, bed nucleus of the stria terminalis, nucleus accumbens/ventral striatum and ventral tegmental area [17-20]. While prior work has identified a network of regions involved in non-painful aversion-related processing [5], it nonetheless remains unclear which, if any, of those identified areas are also involved in processing painful stimuli.

### Systematic translational analysis of aversion-related circuitry

The present hypothesis is that there exists a core aversion-related circuit involved in processing aversive stimuli regardless of whether they are painful or non-painful. In an analogical sense, this network would be similar to the basic underlying (e.g. mesocorticolimbic) circuitry identified in the field of reward [21-23]. Prior meta-analyses in humans have outlined core regions associated with pain processing [6,24], and some animal work has even suggested the existence of an overlapping pain and non-pain-related aversion network [25]. Nonetheless, no investigations have used both human and animal data to directly explore the possibility of a shared network for pain- and non-pain-related processing.

To this end, a translational cross-species approach was used to identify the core components of the potential aversion-related network. More specifically, our first aim was to compare brain activations to the passive reception of painful aversive stimuli in healthy adults (using a meta-analysis of human imaging data; i.e. functional magnetic resonance imaging, fMRI, or positron emission tomography, PET) to those in rodents (using a systematic review of studies including markers of cellular activation and available imaging studies). Secondly, we aimed to compare the results on pain-related processing to those gathered previously on the processing of passive non-painful aversive stimuli in humans (meta-analysis) and animals (systematic review) [5].

Our main hypothesis is that aversive stimuli, regardless of origin (e.g. sensory modality) or perception (e.g. painful or non-painful), are processed largely by a common network of brain regions. However, some areas may be more (or uniquely) involved in different aspects of pain- and non-pain-related aversive processing. The use of a meta-analytical approach allows for the clear distinction of areas which have been identified reliably across numerous studies – in comparison to individual studies which may have low power and a higher probability of reporting false positive activations [26]. The incorporation of animal studies allows for a cross-species comparison and ensures that especially subcortical areas, which may be important for aversion-related processing, are identified. Studying these areas in humans has proven difficult given limitations in optimal imaging resolution and the correct interpretation of subcortical activations (or the lack thereof) [27]. Importantly, this translational approach allowed for the direct comparison of the overlap between areas identified in pain and non-pain aversion studies.

## Results

### Pain-related activation in humans (meta-analysis) and rodents (systematic review)

**Figure 1:**
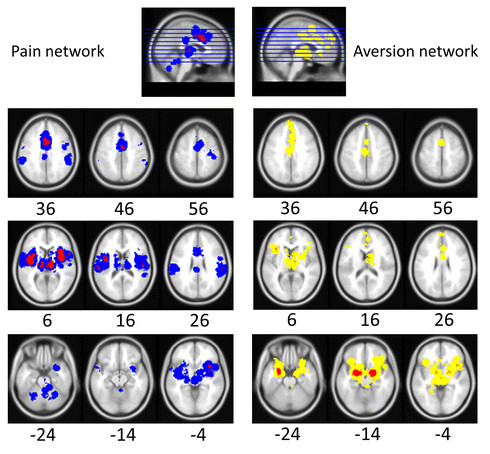
**Pain and aversion networks in humans.** Results of meta-analysis for human pain-related (left) and aversion-related (right) studies. Pain-related (left) activations (see Additional file 1: Table S1A for related coordinates): Red represents peak voxels in a local neighbourhood, blue represents significant extended clusters. Aversion-related (right) activations (see Additional file 1: Table S1B for related coordinates): Red represents peak voxels in a local neighbourhood, yellow represents significant extended clusters. All results are family-wise error rate whole-brain corrected at *p* < 0.05. Numbers below each axial section represent the Z coordinates. The anatomical reference space is MNI 152 (i.e. the average of 152 healthy MRI brain scans). The aversion network was previously reported by [5] and is reprinted here with permission from Frontiers in Integrative Neuroscience.

Results of the meta-analysis revealed a general pain-related brain circuitry involving the bilateral insula, mid cingulate cortex (MCC), postcentral gyrus (primary and secondary somatosensory cortices), precentral gyrus (motor cortex), secondary/supplementary motor area (SMA), and thalamus (Thal). Additional extent-based clusters, extending from regions with peak activations, were also noted in the anterior cingulate cortex (ACC), posterior cingulate cortex (PCC), dorsomedial prefrontal cortex (DMPFC), bilateral operculum, bilateral supramarginal gyri, right ventrolateral orbitofrontal cortex (VLOFC), right rostral temporal gyrus (RTG), right hippocampal/parahippocampal area (Hipp/Parahipp), inferior frontal gyrus, dorsal striatum (DS), cerebellar cortex, and midbrain and rostral pons (Figure 1 and Additional file 1: Table S1A).

As revealed by a systematic review of the non-human animal literature which is summarized in Table 1, all of these areas have also been implicated in animal studies of pain, with the exception of areas corresponding to the supramarginal gyrus and rostral temporal gyrus. Additional subcortical areas, not predominantly found across human studies, have also been noted and include the amygdala, bilateral hippocampus/parahippocampal areas (Hipp/Parahipp), septal area, nucleus accumbens (NAc; a major part of the ventral striatum, VS), bed nucleus of the stria terminalis (BNST), piriform cortex, and retrosplenial cortex, as well as areas of the midbrain (i.e. periaqueductal grey (PAG), superior (SC) and inferior (IC) colliculi, habenula (Hab), raphe nuclei, pretectal area, and red nucleus) and of the brain stem (i.e., nucleus of the tractus solitaries (NTS), parabrachial nucleus (PBN), locus coeruleus (LC)). For individual study details and inter-study comparisons, see Additional file 1: Table S2A.

**Table 1:** Major brain activations in 32 pain non-human animal studies

**Rank order: Pain (32 studies)**		
**Area**	**Studies reporting activation**	**Percentage**
Cingulate	15	47%
Thal	14	44%
Sens	13	41%
Hyp	12	38%
Amyg	9	28%
PAG	9	28%
DS	9	28%
Motor	7	22%
Ins	7	22%
Hipp/parahipp	6	19%
NTS	5	16%
IC	5	16%
LC, PBN, Pretectal area, SC, Septal area	4 each	13%
Hab, NAc, Raphe, Piriform, PFC(IL/PL)/OFC, Visual ctx	3 each	9%
Aud ctx, BNST, Cerebellum, Retrosplenial	2 each	6%
Red n	1	3%

Results are ordered by the number of studies reporting specific activations; percent of studies reporting activations is used for comparative illustration. For individual study details and inter-study comparisons, see Additional file 1: Table S2A.

### Non-pain-related aversive brain activation in humans and other animals

As published previously by Hayes & Northoff (2011), the meta-analysis results of human neuroimaging studies using passive non-painful aversive stimuli implicated brain circuitry involving the amygdala (Amyg), anterior insula (AI), ventrolateral orbitofrontal cortex (VLOFC), hippocampus (Hipp), and parahippocampal gyrus (Parahipp), dorsal striatum (DS), rostral temporal gyri (RTG), and thalamus (Thal). Extent-based clusters were also noted in the anterior and middle cingulate cortex (ACC and MCC), dorsomedial prefrontal cortex (DMPFC), secondary motor area (SMA), and midbrain (Figure 1 and Additional file 1: Table S1B) [5].

Animal studies involving non-painful aversive stimuli implicated all of the same regions shown in humans, except the rostral temporal gyri specifically (see Table 2). In addition, subcortical areas such as the bed nucleus of the stria terminalis (BNST), habenula (Hab), hypothalamus (Hyp), nucleus of the solitary tract (NTS), nucleus accumbens (NAc), periaqueductal grey (PAG), parabrachial nucleus (PBN) and septal nuclei were also noted. For individual study details see Additional file 1: Table S2B.

**Table 2:** Major brain activations in 42 aversion non-human animal studies

**Rank order: Aversion (42 studies)**		
**Area**	**Studies reporting activation**	**Percentage of studies reporting activation**
Amyg	32	76%
Thal	13	30%
Hyp	12	29%
NTS	10	24%
Parahipp/Hipp	9	21%
PBN	8	19%
PAG	8	19%
Ins	7	17%
PFC (PL, IL)/OFC	7	17%
BNST	5	12%
NAc	5	12%
Septal	3	7%
ACC, DR, DS, LC	2 each	5%
Motor, Hab, VTA	1 each	2%

Results are ordered by the number of studies reporting specific activations; percent of studies reporting activations is used for comparative illustration. For individual study details and inter-study comparisons, see Additional file 1: Table S2B. Reprinted with permission from Frontiers in Integrative Neuroscience [5].

### Comparison between pain- and non-pain-related activations in humans and animals

#### Conjunction and contrast analyses in humans

**Figure 2:**
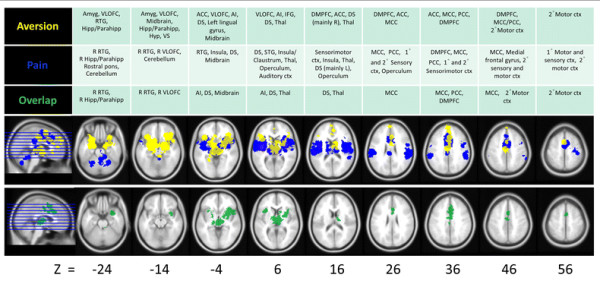
**Overlap of pain- and aversion-related networks in humans.** Results of meta-analyses for human pain- (blue) and aversion- (yellow) related studies (top row), overlapping activations (green; top row and isolated in bottom row), and a corresponding table of associated brain regions. All results are family-wise error rate whole-brain corrected at *p* < 0.05. See Table 3 for overlap coordinates.

A conjunction analysis of the human meta-analysis results for pain- and non-pain-related aversive stimuli revealed a common network of brain areas including: MCC, posterior cingulate cortex (PCC), AI/claustrum, right VLOFC, DMPFC, right RTG, SMA, thalamus, right Hipp/Parahipp, dorsal striatum, and midbrain (see Figure 2, Table 3). We used the conservative minimum statistic resulting in the intersection of 5900 voxels at the cluster-based FWE corrected level [28]. While all clusters are displayed in Table 3, only those greater than 10 voxels were considered in the discussion. The percentage of overlapping voxels in the present study accounted for 24% of pain-related activations and 35% of aversion-related activations.

**Table 3:** Overlap between painful and non-painful aversion-related brain activations in humans studies

**Cluster**	**x**	**y**	**z**	**voxels**	**Volume (mm3)**
1	16	-3	-1	4019	32152
2	1	9	39	1418	11344
3	-33	17	2	400	3200
4	-2	-23	36	20	160
5	7	-38	-21	20	160
6	-21	12	-3	5	40
7	-24	-6	-9	3	24
8	-16	15	-4	2	16
9	4	-25	38	2	16
10	9	-30	-20	2	16
11	-12	18	8	1	8
12	-18	-28	0	1	8
13	8	0	-6	1	8
14	-32	-2	-10	1	8
15	-34	4	-12	1	8
16	-12	12	8	1	8
17	-20	6	4	1	8
18	18	16	0	1	8
19	4	-28	-20	1	8

Results of MKDA analysis: Overlap for painful and non-painful stimuli highlighted in the bottom row of Figure 2. All results are family-wise error rate whole-brain corrected at *p* < 0.05. Only clusters >10 voxels were considered.

In addition to the activation of this common network, the presentation of painful compared to non-painful stimuli (painful > non-painful) resulted in additional unique activations in the primary and secondary sensorimotor cortices, posterior cingulate cortex (PCC), the bilateral operculum (including bilateral SMG), cerebellum, and the rostral midbrain (in a region consistent with the VTA; see [29]) and rostral pons (see Figure 2).

Alternately, non-painful > painful aversive stimuli resulted in unique activations in the amygdalae and left hemisphere VLOFC, RTG, and Hipp/Parahipp. In addition, extended activation clusters unique to non-painful stimulus presentation were noted for the more anterior portions of the ACC (pregenual and subgenual ACC), the DMPFC, a more anterior portion of the PCC/posterior MCC, an area encompassing the hypothalamus and dorsal (DS) and ventral striatal (VS) areas, left SMA, and bilateral VLOFC (Figure 2).

#### Comparison to non-human animals

While a conjunction analysis was not performed across non-human animal studies (given that precise coordinates related to neural/cellular activation are less often given), the pattern of activation seen in humans was reflected in these studies. The general pattern of brain activation between humans and animals appears to be maintained, as is generally reflected by the relative percentage of studies reporting such activations (see Tables 3 and 3). For instance, a large percentage of animal studies have reported cingulate activations in pain-related studies (Table 1) which is mirrored by the large activation in the pain (but not the non-pain) meta-analysis; in comparison, a majority of animal studies have reported amygdala activations in response to non-painful aversive stimuli (Table 2), which is reflected by the large activation in the non-pain (but not the pain) meta-analysis results.

**Figure 3:**
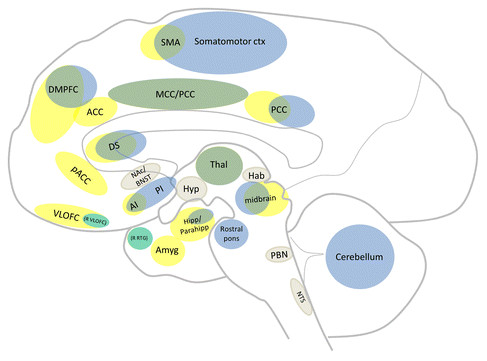
**Similarities and differences in pain- and non-pain-related aversive circuitry.** Sagittal section of a human brain summarizing the main results across species; illustrating core areas consistent with responses to all pain- and non-pain-related aversive stimuli (green), those responding to painful (blue) or non-painful (yellow) aversive stimuli alone, and regions implicated mainly in non-human animal studies (beige).

Additional subcortical regions of importance (which may be too difficult to detect or differentiate precisely using human brain imaging techniques) have also been noted in animals (see Tables 1, 2). Most seem to be involved in general aversion-related processing (i.e. BNST, Hab, Hyp, LC, NAc, NTS, PAG, PBN, septal area, anterior raphe nuclei) while this investigation suggests that some may be involved more in pain- (i.e. IC, SC, pretectal area, red nucleus, retrosplenial area) or non-pain-related (i.e. basolateral and central nuclei of the amygdalae, VTA) processing. Results for overlapping and non-overlapping activations across species are illustrated in Figure 3.

## Discussion

The present work aimed to further define a network of brain regions involved in the general processing of aversive stimuli (both pain- and non-pain-related) using a cross-species translational approach. A direct comparison was made between studies investigating the passive reception of painful versus non-painful aversive stimuli in humans (using a meta-analysis; Figures 1 and 2) and rodents (using a systematic review; Tables 1 and 2).

Firstly, it was found that regions associated with the processing of painful aversive stimuli (Figure 1, Additional file 1: Table S1A) and those noted previously with non-painful aversive stimuli (see [5]; Figure 1, Additional file 1: Table S1B) are similar across humans and other animals (Tables 1,2). The value of including animal data is that they help support and extend the results noted in humans (see further discussion below). Secondly, it was shown in humans (Figure 2, Table 3) and other animals (Tables 1 and 2, also see Additional file 1: Tables S2A and Additional file 1: Table S2B) that most regions show spatially overlapping involvement for the processing of both painful and non-painful aversive stimuli.

Together, these data strongly suggest the existence of a core aversion-related network of brain regions which include cortical (i.e. MCC, PCC, AI, DMPFC, RTG, SMA, VLOFC; see results section or abbreviation section for full names) and subcortical (i.e. Amyg, BNST, DS, Hab, Hipp/Parahipp, Hyp, NAc, NTS, PAG, PBN, raphe, septal nuclei, Thal, LC, midbrain) areas. Lastly, although a core aversion-related network was identified, both painful and non-painful stimuli activate additional regions which appear to be non-overlapping, although this is discussed further below (summarized and illustrated in Figure 3).

### Areas activated by painful and non-painful aversive stimuli are similar across species

Results from the pain-related meta-analysis (Figure 1, Additional file 1: Table S1A) and animal literature review (Table 1, Additional file 1: Table S2A) are consistent with prior human [30,31] and animal studies [32,33]. Similar regions were noted here, including: bilateral insula, MCC, primary and secondary motor and somatosensory cortices, VLOFC, Hipp/Parahipp, DS, Thal and midbrain. It is also worth noting that our results are nearly identical to another recent human meta-analysis on pain [24]. Additional, mainly subcortical, areas identified across animal studies included the Amyg, BNST, Hab, Hyp, NAc, NTS, PBN, PAG, SC, IC, and septal nuclei. Brain imaging techniques may be limited by low subcortical resolution (see [34] for a brief discussion of fMRI capabilities), however some fMRI studies focusing on this level (e.g. PAG, PBN, VTA) have corroborated their involvement in human pain processing (e.g. [35]).

As reported previously [5], non-painful aversive stimuli result in consistent activations in humans (Figure 1, Additional file 1: Table S1B) and other animals (Table 2, Additional file 1: Table S2B). The main activations to non-painful aversive stimuli across species include: Amyg, ACC, VLOFC, DMPFC, secondary motor cortex, Hipp/Parahipp, DS, RTG, Thal, and midbrain. Additional subcortical activations, identified most consistently in animal studies (although see human imaging studies for instance by [36] and [37]), included the BNST, Hab, Hyp, NTS, NAc, PAG, PBN and septal nuclei. These results are also consistent with prior studies across species looking at aversion-related concepts such as fear [38,39], threat [40,41] and social punishment [42,43]).

Together, these findings suggested the involvement of similar regions in pain- and non-pain-related aversive processing (Figure 1). However, the specific substrates involved remain unclear as does whether the apparent spatial overlap involved in both processing types reflects an actual overlap in function. Iannetti and Mouraux (2010) have argued that the so-called Pain Matrix (reflecting a pain-specific network of brain regions; see [44,45] for early uses of the term) may be misleading. While the Pain Matrix is activated reliably across studies (as underscored here and in other meta-analsyses, e.g. [24]), and the magnitude of its response is highly correlated with the intensity of pain perception [46,47], there is, nonetheless, presently no evidence that any single brain region or clearly defined network is devoted to the processing of pain.

Iannetti and Mouraux (2010) (and other authors, e.g. [13]) subsequently suggested that, instead of pain-specific processing, this system may be better regarded as a multimodal network related to the detection of saliency [11]. The notion that this network is largely multimodal is supported by prior work demonstrating activations in these regions regardless of the sensory modality in which the aversive stimuli were presented [5,12]. As well, this notion is further supported by the present findings that painful stimuli (which are all tactile in nature) show overlapping activations with mainly non-tactile, non-painful, aversive stimuli. In particular, in a series of fMRI experiments, Mouraux et al. (2011) showed that most of the activations related to painful stimuli appear to also be involved in non-painful processes [12].

In another recent example, fMRI work by Moulton et al., (2011) showed similar overlapping between activations related to non-painful (i.e. unpleasant pictures) and painful (i.e. noxious heat) aversive stimuli in the cerebellum; these signals were correlated with many of the same regions noted in the present study (e.g. Hyp, ACC, Hipp/Parahipp) [48]. Interestingly, it is difficult to assess the cerebellum’s potential involvement in general aversion-related processing given that it is often overlooked in imaging studies and most animal studies use painful shock-tone pairing to investigate its role. As such, while its role in general conditioning mechanisms is well established (e.g. [49]), fewer studies have looked at non-pain-related aversive stimuli (though note research showing its involvement in conditioned taste aversion; e.g. [50]).

As the frequent occurrence of similar brain activity appeared to be further supported by the initial cross-species results above, the second aim of this study was to specifically compare the potential overlap between pain- and non-pain-related activations in humans and other animals.

### Aversion-related stimuli activate a common core network

The conjunction analysis of pain- and non-pain-related aversion studies in humans (Figure 2, Table 3) and the systematic review in animals (Tables 1 & 2; Additional file 1: Tables S2A & S2B) revealed a common core aversion-related network consisting of MCC, PCC, AI/claustrum, right VLOFC, DMPFC, SMA, right Hipp/Parahipp, Thal, DS, and midbrain (including an area encompassing the PAG). Additional, mainly subcortical, areas were noted in animals including the Amyg, BNST, Hab, Hyp, NAc, septal nuclei, NTS, and PBN. This core, cross-species, aversion-related network (illustrated in Figure 3) suggests that passively received aversive stimuli are, at least in part, processed in a similar way using similar neurobiological substrates.

Identified consistently here, both the cingulate (especially the MCC) and bilateral AI have been implicated in many functions though there is still much debate on whether a basic role for these regions exists. For instance, research in humans and other animals suggests they are involved in detecting and processing errors [51,52], in reward-related processing [53,54], and in adaptive decision making [55,56]. In fact, given the roles that these, and other, regions play across various networks (e.g. salience, interoceptive, resting state, and valuative), it is possible that they are active participants in many underlying processes. For instance, various lines of evidence in humans and primates have implicated the anterior portion of the MCC as being especially key for the integration of negative affect, pain, and cognitive control (as reviewed in [57]). Moreover, despite our attempt to reduce the impact of cognitive processes by limiting our studies to those involving passive stimuli, it is important to note that implicit cognitive processing (e.g. brain activity related to preparatory escape, emotional regulation) may contribute to the activations noted across species.

#### Involvement in salience network

For instance, it has been suggested that main role of the ACC might be learning and predicting the outcome of actions, regardless of valence [58] – essentially a processor and predictor of salient/behaviourally relevant sensory stimuli. A human imaging meta-analysis on the role of the insula also suggested that the AI may be an integrator of salient stimuli [59]. The notion that the cingulate and AI are involved in salience processing is also in line with the experimental findings of Mouraux et al. (2011) and Downar et al. (2003) [12,13], as is the involvement of the PCC [60,61] and the somatosensory cortex (though the present meta-analysis suggested this region was selective for painful stimuli). They found that the BOLD signal in these regions corresponded best to the saliency of the aversive stimulus, and not whether it is painful or non-painful (using stimuli across sensory modalities). Indeed, the indication that an upcoming stimulus will be painful (i.e. predictors with high salience) has been found to potentiate signals in traditional pain-related regions (e.g. MCC) [62,63]. Along with resting state data showing that the AI is functionally connected to the pACC/MCC [64], these studies strongly support the notion of the cingulate (particularly the MCC) and AI as being involved in salience processing [12,56].

Other regions identified in the present study may also be involved in processing salient stimuli, particularly in the preparation and/or initiation of motor control. Structures such as the DS and SMA appear to process preparatory defensive actions (and/or inhibit unwanted responding) related to escaping potential threats [65]. In general, however, the DS responds to the expected value of stimuli (including reward, e.g. [66]; similar to the cingulate and insula) and may be important in changing expectations based on past and present contexts [67]. The PAG also responds to highly salient (particularly aversive) stimuli, is a well-known source for descending control over spinal pain pathways [68], and appears to be involved in autonomic-somatomotor integration related to orchestrating defensive behaviours [69]. This integration is achieved via its close connections to Thal, Hyp, AMYG, PFC, and other brain stem nuclei involved in autonomic processing (e.g. NTS, PBN, raphe; as noted across the present animal data) [70]. (For a comprehensive review on the PAG in neuroimaging studies see [71].) Considered together, the cross-species data noted here suggest that the core regions in general aversive processing may be involved in processing salience information. This is in line with the findings from Mouraux et al. (2011), who suggested that these regions form a salience (as opposed to a pain) network [12].

#### Involvement in interoceptive network

The AI and ACC/MCC are also considered key components of a circuit mediating interoceptive awareness. Studies have shown that both the insula and ACC/MCC are involved in processing the interoceptive awareness of stimuli (e.g. heartbeat, respiration; e.g. [72]), while animal studies have demonstrated similar roles (e.g. [73]). Menon and Uddin (2010) take a network perspective describing these regions as core components of a salience network which act as integrators of overlapping networks (carrying information related to interoception, homeostasis, working memory, higher-order control processes) [74]. Additionally, the authors posit that the activity of these hub regions and the interaction between them may be involved, partly, in choosing/switching between relevant task-related and resting state networks (e.g. engaging task-relevant memory and attentional processes while disengaging from non-task-related activity). The notion of the ACC/MCC and insula as a key integrator network is also supported by others (e.g. [64]) who have noted functional connectivity between AI-pACC/MCC (which they suggest may integrate interoceptive and emotional information) and between insula-MCC (which may be more involved in exteroceptive processing and response selection).

#### Involvement in resting state networks

The baseline activity of the brain is essential in determining the relative stimulus-induced activation in both animal and human studies. As such, how the baseline is defined conceptually and experimentally may ultimately affect the results (see [75,76] for further discussion). As noted in the Methods section, most of the animal studies included here necessarily use between-subjects measures, whereas the human neuroimaging studies typically use within-subjects measures – suggesting that the baselines may be quite different. As such, caution should be taken when interpreting the results across species. Nonetheless, the animal neuroimaging studies included here (e.g. [77,78]) are able to help bridge the gap somewhat and their findings are congruent with the other studies. Moreover, human imaging studies are more often including a baseline (or so-called resting state) period in their designs (in which the subject is instructed to stare at a fixation cross or close their eyes and not focus on any particular thoughts), which results in the identification of baseline or resting state brain activity.

Given the apparent overlap and interactivity between these resting state regions (e.g. VMPFC/pACC and PCC) and exteroceptive/salience regions, some authors have recently explored their potential relationship. For example, one recent study investigated the potential relationship between resting state activity (within the default mode network) and emotion- and intero-/exteroceptive-related activity [79]. They demonstrated that increased activity in the default mode regions (e.g. VMPFC/pACC, PCC) during rest was associated with decreased emotional perception ability, without any noted relationships to the perception of intero-/exteroceptive stimuli. This raises the question of whether the activity, particularly in the MCC, is selective for salience or rather for value-related processing (related to determining the positive or negative value of a stimulus). Moreover, it questions whether different regions of the cingulate (or similar regions activated differentially across time) are involved separately in processing salience-, emotion-, and interoceptive-related information (e.g. [64]).

#### Involvement in valuative network

Finally, recent reviews of the reward literature for both humans [80] and animals [23] describe many of the same regions noted here for the aversion-related network. This raises the issue of whether, and to what degree, the core aversion-related regions noted in the present study are involved in aversion-specific (not reward-related) processing. If both aversion- and reward-related activity are found equally in these regions, this would provide further support of a salience network. However, if different regions are involved in each, and/or to different degrees, this would suggest the existence of interacting, or even separate, neural networks for processing value-related information. While there are, to our knowledge, no meta-analyses or systematic reviews outlining the similarities and differences for reward- and aversion-related brain activity, a number of studies (particularly at the neuronal level) have suggested that perhaps both salience-selective and value-selective processing occur in both overlapping and separate networks.

For instance, many animal (e.g. [81]; and see [20] for review) and some human studies (e.g. [36,37] and see [82] for review) have implicated the NAc in coding both aversive and rewarding states. Lammel et al. (2011) showed that among dopamine cells of the VTA there appear to be many distinct (reward-, aversion-, or saliency-related) populations defined by receptor/channel type, activity, location and density, and axonal projections [83]. Another study demonstrated that while the dopaminergic modulation of the basal ganglia’s direct striatonigral and indirect striatopallidal pathways are involved in both processing types, primary activation is shifted to the indirect pathway during aversion-related processing [84]. Furthermore, single-unit ACC recordings in four humans viewing emotional pictures showed that of cells responding, most were selective for general aversion-related stimuli – although some cells responded to both aversive and rewarding stimuli [85]. These findings help explain how a regional population of cells can contribute to differential processing (e.g. aversion and reward).

Taken together, these results support the existence of a core group of brain regions involved in basic aversion-related processing (Figure 3); the inclusion of non-human animal data has helped confirm and further extend these findings (mainly regarding subcortical regions). The evidence suggests that components of this network are involved in processing various types of information (e.g. saliency, interoception, valuative). Future neuroimaging studies should consider parsing, for instance, value and salience (e.g. using multi-levelled stimuli, to investigate ‘dose–response-like’ curves) as has been attempted in some behavioural experiments [86]. Though the focus has been on the overlap between pain- and non-pain-related aversive processing, it should be noted that some activations (particularly in the meta-analysis) appeared to be selective for painful over non-painful stimuli (and vice versa). These activations are discussed briefly in the following section.

### Differential activations related to painful and non-painful aversive stimuli

Although a core network of brain regions for the processing of painful and non-painful aversive stimuli was identified, the areas of activation noted in the human meta-analysis do not overlap completely. In fact, there are a number of regions which appear to be unique to painful or non-painful stimuli. Specifically, painful stimuli resulted in unique activations in the primary and secondary sensory cortices, the PCC, bilateral operculum/mid and posterior insula, and cerebellum. Non-painful aversive stimuli resulted in unique activations in the AMYG, L VLOFC, L RTG, and L Hipp/Parahipp as well as additional extended activations in the pACC, DMPFC, HYP, posterior MCC, DS/VS, and bilateral VLOFC. Similar subcortical regions were identified in animal studies of both pain- and non-pain-related processing (e.g. NTS, PBN, BNST). Nonetheless, from the animal studies included here, a few areas are noted in only pain- (i.e. red nucleus, pretectal area, IC/SC) or non-pain- (i.e. VTA) related studies.

The differences may in fact be unique, thus providing unique neural signatures for painful and non-painful aversive stimuli. However, there are alternative explanations for these apparently unique activations which seem more tenable. Firstly, the differential weighting of activations (i.e. that some regions are more involved) may help explain the absence of activations in human studies on pain (e.g. amygdala) compared to non-pain aversion. Secondly, as the analysis of imaging studies on pain and non-pain aversion were limited to the passive reception period, this is equivalent to taking a temporal snapshot of brain activity. While intended to be similar across pain and non-pain studies, this snapshot may in fact reflect unique brain processing for two types of stimuli differentiated by their timing properties (i.e. painful stimuli result in fast aversive responding; non-painful stimuli are slower and more variable).

#### Differential weighting

That nearly all regions were noted in both animal studies of pain and non-pain aversion (Tables 1 and 2; e.g. involvement of the amygdala, [32,87]) supports the notion that entirely unique activations for either are rare or unlikely. However, a differential weighting of activations may be reflected in the ranking of regions in animal studies (Tables 1 and 2), which generally reflect the core regions noted in the human meta-analysis (Figure 2). For instance, the amygdala is activated more consistently in non-pain imaging studies, whereas the cingulate and sensory cortex appear more involved in pain studies (Figure 1). However, these rankings (i.e. the percentage of animal studies noting specific brain activations) should only be considered illustrative, as animal studies typically choose regions a priori (compared to a whole-brain approach). Findings for a set of regions may lead to a disproportionately higher investigation rate by other researchers, which can inflate or mask the relative importance of some regions (though brain imaging is also not immune to such biases; see [88] for a brief discussion of this in relation to meta-analyses). Nonetheless, most animal studies included here investigated ≥5 brain regions, and the results are similar to those in humans and other animals (including studies investigating other aversion-related concepts such as fear [39], threat [41] and social punishment [43].

#### Differential temporal dynamics

Another potential explanation for differences between pain and non-pain aversion includes the timing of activity, as noted above. While the temporal dynamics of this circuitry have not been worked out, conditioning studies in animals [89,90] and humans [91,92] have suggested temporal and/or subregional differences between the processing of conditioned stimuli predicting an aversive stimulus and the reception itself (underscoring the importance of spatio-temporal dynamics). For instance, the amygdala is known to be more involved in assessing the expectation (especially involving the timing) of aversive stimuli [93,94] – though see [95] for an fMRI study in which long stimulation periods of pain perception resulted in amygdala activation. Another study by Guimarais et al. (2011) showed that increasing the time interval between a predictive tone and a shock changed the involvement of some structures in rats [96]. For instance, at ~5 s intervals, the mPFC became more active, whereas at longer (~40s) intervals, dorsal hippocampal activity became necessary for learning about the aversive stimulus. Nonetheless, it is difficult to compare the results from animal studies directly to those in human imaging (especially without direct translational mapping), and further study on the temporal aspects of aversive processing should be undertaken.

### Strengths and limitations

The greatest strength of the current work is its translational nature. The inclusion of animal studies has two main advantages. Firstly, they help to support the findings from human imaging and add insight regarding subregional differences and underlying mechanisms. Secondly, they underscore the involvement of many subcortical regions which are generally underreported in imaging studies (as noted previously). We believe using this approach somewhat offsets the potential selection biases which may be found across human imaging studies (e.g. lowering statistical thresholds for a priori regions) and animal studies (e.g. looking only for activity in a priori regions; also see below).

The strict and narrow criteria used in both human and animal studies allowed for a clearer interpretation of results (e.g. the use of passive and acute aversive stimuli only; the exclusion of studies/subjects using explicit cognitive tasks; see Methods section for all criteria). These criteria were used to isolate, as clearly as the present methods allow, the period of brain activation during which acute aversive stimuli are present (e.g. most neuroimaging studies look at periods around 5–10 seconds; the animal studies included here extract the brains as soon as possible following stimulus presentation) – thus, attempting to separate this period from others (e.g. anticipation, termination). It is in this sense that we have attempted to identify a network associated with aversion-related processing (see also [26] for further discussion on using meta-analyses to identify functionally related brain regions). Nonetheless, it is worth pointing out again (as discussed briefly above in the Differential weighting and Differential temporal dynamics sections) that the inference of a temporal relationship between regional activations relies heavily on the inclusion, and exclusion, of appropriate studies. Ultimately, the identification of such networks through meta-analyses and systematic review should be used as the basis for testing future hypotheses regarding co-activation.

This approach also shed some light on one inherent and important limitation of meta-analyses – particularly those using human imaging studies. While some neuroimaging studies do report subcortical activations in aversion-related processing (e.g. [37,97,98]), their relative scarcity means some subcortical regions may not be noted in the final meta-analysis results. This absence of activation likely also extends to highly variable cortical regions. The corollary is that meta-analysis results underscore the most consistent nodes of activation across studies (with the coordinates being more informative than the size or shape of the clusters per se; see also Differential weighting discussion above), while regions not identified may still be active (and even essential) components – findings that are made clearer through animal studies looking directly at brain tissue.

Although the results of the animal studies outlined in Tables 1 and 2 (i.e. listing the percentage of reported brain activations) should be considered illustrative due to reporting and researcher interest biases and the lack of a whole-brain approach (as insisted upon for the imaging data), most studies investigated at least 5 brain regions. In fact, only 9 of the 42 non-pain-related studies [89,90,99-106] and 13 of the 32 pain-related studies [87,107-118] focused on 4 or less regions. In addition, none of the pain-related studies focused solely on the cingulate (a key node identified in both human and animal data), and only 3 studies from the non-pain-related aversion studies [100,105,106] focused solely on the amygdala (perhaps the single best described aversion-related region). Although the issue of selection bias and the reporting of positive data (the so-called file-drawer problem) cannot be fully accounted for, taken together, the animal and human data allow for a more confident interpretation regarding the inclusion of brain areas involved in aversion-related processing.

## Conclusions

The results from this translational approach strongly suggest that humans and animals have a common core aversion-related network, consisting of similar cortical and subcortical regions. This work extends from previous work [5] by demonstrating that most of the regions typically associated with a pain network are involved in the generalized processing of all aversive stimuli to some extent. While saliency may be an integral factor to which this network responds, it seems unlikely that it should be thought of as a saliency detector – particularly given the apparent incomplete overlap of activations for painful and non-painful stimuli, as well as for that of rewarding stimuli. The differential weighting found between pain- (e.g. higher activations in MCC and posterior insula) and non-pain- (e.g. amygdala) related processing suggests that aversion-related concepts may rely on the use of similar substrates (i.e. aversion-related network) but to varying degrees and perhaps over different timescales – thus underscoring the need for the investigation of spatio-temporal dynamics within this network.

## Methods

### Painful and non-painful aversion-related brain activation in humans

Literature search: We identified all imaging studies – positron emission tomography (PET) and functional magnetic resonance imaging (fMRI) – published from 2000 to August 2011 with PubMed (http://www.pubmed.gov) and Web of Science (http://apps.webofknowledge.com; though no additional studies were found here) searches. Keywords included "aversion", "aversive", “avoidance”, “punishment”, “reinforcement” (to capture some studies focusing on reward, but also using an independently analysed aversive control condition), “fear”, “anger”, “disgust”, “sadness”, “negative emotion”, “pain”, “nociceptive”, “unpleasant”, "positron emission tomography", and "functional magnetic resonance imaging" and others. Furthermore, we searched the reference list of articles and reviews, including meta-analyses (for example see [6,119,120]). The data regarding brain activations associated with non-painful aversive stimuli were reported previously in [5].

Inclusion and exclusion criteria: Our main goal was to compare the basic brain activity of painful- and non-painful aversive stimuli. Therefore, we included only those studies which used the passive presentation of acute aversive stimuli (e.g. the viewing of unpleasant pictures; exposure to painful stimuli) without active responses. Without behavioural measures, aversive experiences were determined subjectively and often supported through physiological measures such as electrodermal activity. Designs whose contrasts did not include specific comparisons relevant to the current analysis (i.e. involving the passive reception of painful or non-painful aversive stimuli, independent of explicit cognitive processes, memory, or attention) were excluded. In addition, only studies that reported coordinates from whole-brain analysis were included (although some studies discussed region-of-interest data, those coordinates were not included here). These criteria lead to the inclusion of studies involving both exteroceptive (e.g. pictures, shock) and interoceptive (e.g. rectal distension) aversive stimuli.

Although the related search terms were included for completeness, studies reporting the responses to specific negative emotions (e.g. sadness, anger) were excluded given their social nature as were other stimuli which may involve ambiguous interpretations such as those involving empathy, disgust, or physical contamination. For related examples involving such analyses showing results consistent with the present study, see [121,122]. Other studies looking explicitly at social aspects of aversion, such as social exclusion, were also excluded given that they typically require complex behavioural responses and involve other potentially confounding processes such as empathy and theory of mind. Moreover, it is important to note that most studies involving conditioned fearful stimuli in both humans and animals were excluded to avoid issues related to recent learning effects and complex conditioning designs, although it should be noted that the aversive stimuli used are virtually identical to those included here and the results from such studies are largely similar to those reported here [123,124]. Finally, studies involving manipulations in homeostatic states (e.g. hunger, thirst) were also not included given their non-acute nature (e.g. usually involving forced deprivation over time). Although we aimed to cast as large an initial net as possible for both the human and other animal studies, we must concede that, given the conceptual complexity of aversion-related terms, we may have overlooked some studies which do not employ standard terminologies.

We screened all the articles for Talairach or Montreal Neurological Institute (MNI) coordinates and tabulated the reported regional foci. We included the data of healthy subjects only. Studies including individuals with psychiatric illnesses, or a history thereof, those with volumetric abnormalities or brain injuries, those taking any medications or illicit drugs, and those belonging to a group that may result in a sample bias (e.g. war veterans) were excluded. In addition, a large number of studies were excluded due to the absence of coordinates, identification of coordinate systems, and/or incomplete statistical information. These criteria resulted in the selection of 34 non-pain-related studies which included 44 contrasts, and 27 pain-related aversion studies which included 32 contrasts (see Additional file 1 for study references included in the meta-analysis). Regions were labelled macroanatomically by the probabilistic Harvard-Oxford atlas. The nomenclature for the cingulate by Vogt (2005) was used here [125].

Multilevel kernel density analysis (MKDA) meta-analytic technique: The MKDA meta-analytic approach has been covered in depth elsewhere [26,126]. Briefly, MKDA is a coordinate-based meta-analytic method which determines the activation probability of each voxel and contiguous voxel clusters (to create voxel-based and cluster-based study comparison maps, respectively) across the brain. Compared with other meta-analysis methods, MKDA prevents any single study reporting a large number of activations from biasing the results (i.e. the study is the unit of analysis) and weights contrasts based on the quality of the study (e.g. random vs. fixed effects) and the sample size. It should be noted that study-specific statistical thresholding is not taken into account, however, because weighting activation peaks by their respective Z-scores introduces the presupposition that the scores across studies are comparable, which is untrue (e.g. scores from studies with small sample sizes would be inflated relative to the larger population; [26] for further discussion and examples). All results are reported in MNI space; co-ordinates from studies using Talairach space were converted to MNI space in the MKDA software using the Brett transform (http://imaging.mrc-cbu.cam.ac.uk/downloads/MNI2tal/tal2mni.m). Peaks from each study were convolved with a spherical kernel of 10 mm radius; doing so ensures that multiple nearby peaks are not counted as multiple activations (additionally helping to reduce the impact of studies employing lower statistical thresholds and which typically report a greater number of activated voxels). This approach also ensures that single studies do not drive the results of the meta-analysis. The threshold for significance was determined using a Monte Carlo simulation with 3000 iterations (5000 iterations did not alter the results) and a null hypothesis which assumes that the activated regions within each map are not spatially consistent (i.e. that the cluster centers are randomized throughout the grey matter). The voxel size was 2 x 2 x 2 mm (i.e. 1 voxel = 8 mm^3^) and cluster sizes were all greater than 10 voxels (> 80 mm^3^). We have reported peak voxel-wise activations as well as peak cluster-wise activations (which include contiguous voxels significant at *p* < 0.001; whole-brain FWE corrected). All results are family-wise error rate whole-brain corrected at *p* < 0.05. Analyses were performed in Matlab 2009a (Mathworks, Naticks, MA) using MKDA software created by Tor Wager (http://www.columbia.edu/cu/psychology/tor/).

### Painful and non-painful aversion-related brain activation in animals

Literature search: PubMed (http://www.pubmed.gov) and Web of Science (http://apps.webofknowledge.com; again, no additional studies were found here) searches identified rodent studies related to pain-related (~700 studies) and non-pain-related (~350 studies) aversion published in English from 2000 to August 2011. Keywords were similar to those above but also included specific terms related to animal studies such as: “immediate early genes”, “IEG”, “c-fos”, “rat”, “mice”, “monkey”, “mammal”, “avoidance”, “fear”, “threat”, “electrophysiology”. Of the total studies identified, only those clearly showing altered brain metabolism (e.g. increased/decreased c-Fos or blood oxygenated level dependent activity, or BOLD) were included in the systematic review (i.e. 34 pain and 42 non-pain aversion studies). Due to lack of methodological instruments, absence of precise standardized coordinate systems, and the wide range of experimental procedures, we did not conduct the same rigorous meta-analysis in animals as in humans. As with the human data, the non-human animal data regarding the presentation of passive non-painful aversive stimuli were reported previously in [5].

Inclusion and exclusion criteria: We looked at the following metabolic indexes of non-human animal brain activity: immediate early gene activation (e.g. c-Fos or Fos-like expression), BOLD activity in fMRI, [14 C]-2-deoxyglucose, and [14 C]-iodoantipyrine. Each of these indexes has previously been related to neural activity and/or metabolism. Considering the broad spectrum of animal models of aversion-related behaviour (e.g. formalin-induced nociception, foot shock, conditioned taste aversion etc.), we looked at all those data that report clear effects in brain activity between control animals and those exposed to painful and/or non-painful aversive stimuli. It is important to note that some methods must necessarily use between-subjects measures (e.g. counting IEG-stained cells in brain slices) whereas others use mainly within-subjects measures (e.g. neuroimaging).

Studies involving adolescent animals, chronic exposure to aversive stimuli, and exposure to drugs of abuse and those having a direct effect on aversion- or reward-related brain circuitry, were excluded (although non-drug-exposed controls were included where appropriate). This was done in order to avoid confounding issues related to neurodevelopment and drug interactions and/or drug-induced changes in brain structure or function unrelated to the acute aversive treatment. Studies using electrical/chemical lesions or other irreversible alterations (e.g. the use of knock-out or transgenic rodents, animals bred for psychiatric disorder-related phenotypes; although one study using rats bred for high or low anxiety levels, though not pre-exposed to anxiolytic stimuli, was included [127] as the results following an aversive probe largely converged regardless of anxiety levels) were also excluded for clarity.

Any comparison between human and animal data raises the question of homology of brain regions. Since they show analogous anatomy, comparisons of subcortical regions are not often an issue (see also [128]). In contrast, the issue of homology becomes more problematic in the case of cortical regions that show both anatomical and terminological differences between humans and animals. Nonetheless, even areas which may be considered largely ‘higher-order’ or evolutionarily more recent, such as the prefrontal cortex, may show strong structural and functional homologies between primates and other mammals, such as rodents [129,130]. Concerning cortical regions, we relied on criteria of homology as established by various authors [131-133]. In addition, it should be noted that due to the strict inclusion/exclusion criteria nearly all of the animal studies involve rodents with the exception of one study using monkeys [102]. As such, the generalizability to other mammals should be considered cautiously. Nonetheless, beyond the studies included here, studies involving primates were also considered in the discussion to add support to the translatability of the results (e.g.) [53,66].

## Abbreviations

ACC, anterior cingulate cortex; Amyg, amygdala; AI, anterior insula; Aud, auditory ctx; BG, basal ganglia; BNST, bed n of the stria terminalis; Ctx, cortex; D, dorsal; DMPFC, dorsomedial prefrontal ctx; DS, dorsal striatum; Hab, habenula; Hipp, hippocampal area; Hyp, hypothalamus; IC, inferior colliculus; IFG, inferior frontal gyrus; IL, infralimbic ctx; Ins, insula; Lat, lateral; LC, locus coeruleus; MCC, mid cingulate ctx; N, nucleus; NAc, nucleus accumbens; NTS, n of the solitary tract; OFC, orbital frontal ctx; PAG, periaqueductal gray; Parahipp, parahippocampal gyrus; PBN, parabrachial n; PL, prelimbic; PFC, prefrontal ctx; SC, superior colliculus; Sens, sensory ctx; Sep, septal n; SMA, secondary motor area; SMG, supramarginal gyrus; STG, superior temporal gyrus; Thal, thalamus; VLOFC, ventrolateral orbitofrontal ctx; VTA, ventral tegmental area.

## Competing interests

The authors have no competing interests to declare.

## Authors’ contributions

DJH performed the analyses and drafted the manuscript. DJH and GN conceived of the study, and read and approved the final manuscript.

## Supplementary Material

Additonal file 1 **Table S1A.** Painful aversion-related brain activations in human studies. **Table S1B**. Non-painful aversion-related brain activations in human studies. **Table S2A**. Painful aversion-related brain activations in animal studies. **Table S2B**. Aversion-, non-painful, related brain activations in animal studies [32,33,41,73,77,78,87,89,90,99-103,105-110,113-118,127,134-230]. Click here for file
