# Supplementary material for: Common brain activations for painful and non-painful aversive stimuli
Source: BMC Neurosci. 2012 Jun 7;13:60. doi: 10.1186/1471-2202-13-60 (PMC3464596; doi:10.1186/1471-2202-13-60)
Supplement: Additonal file 1 — Table S1A. Painful aversion-related brain activations in human studies. Table S1B. Non-painful aversion-related brain activations in human studies. Table S2A. Painful aversion-related brain activations in animal studies. Table S2B. Aversion-, non-painful, related brain activations in animal studies [32,33,41,73,77,78,87,89,90,99-103,105-110,113-118,127,134-230]. [file 1471-2202-13-60-S1.doc]

**Abbreviations:** ACC (anterior cingulate cortex); Amyg (amygdala); aud (auditory ctx); BLA (basolateral amygdala); BNST (bed n of the stria terminalis); CeA (central n of the amygdala); CP (caudate-putamen); ctx (cortex); d (dorsal); dACC (dorsal anterior cingulate ctx); DG (dentate gyrus); DRN (dorsal raphe n); GP (globus pallidus); Hab (habenula); Hipp (hippocampal area); Hyp (hypothalamus); IC (inferior colliculus); IL (infralimbic ctx); INC (interstitial nucleus of Cajal); Ins (insula); IPN (interpeduncular nucleus); lat (lateral); LC (locus coeruleus); LH (lateral hypothalamus); M1 (primary motor ctx); M2 (association motor ctx); MeA (medial n of the amygdala); MDT (mediodorsal thalamus); med (medial); MRN (median raphe n); n (nucleus); NAc (nucleus accumbens; part of the ventral striatum, VS); NST (n of the solitary tract); OFC (orbital frontal ctx), PAG (periaqueductal gray); PBN (parabrachial n); PL (prelimbic); PFC (prefrontal ctx); PVT (paraventricular n of the thalamus); RN (red nucleus); S1 (primary sensory ctx); S2 (association sensory ctx); SC (superior colliculus); SN (substantia nigra); Thal (thalamus); V1 (primary visual ctx); V2 (association visual ctx); VAN (ventral anterior n thal); VLN (ventrolateral thalamic n); VMN (ventromedial thalamic n); VPL (ventroposteriolateral thal); VPM (ventroposteriomedial thal); VTA (ventral tegmental area)

**Supplementary Table 1A: Painful aversion-related brain activations in human studies**

| **Table 1A** Pain network in humans  Results of MKDA analysis: Peak voxel activations (upper panel) and peak extended clusters  (lower panel); See Figure 1 for associated activations | | | | | | |  |
| --- | --- | --- | --- | --- | --- | --- | --- |
| **Cluster** | **x** | **y** | **z** | **voxels** | **Volume (mm^3^)** | **Brain Region** | **BA** |
| 1 | -40 | -2 | 8 | 698 | 5584 | L Insula |  |
| 2 | 36 | 8 | 4 | 625 | 5000 | R Insula |  |
| 3 | 0 | 8 | 38 | 411 | 3288 | MCC |  |
| 4 | 10 | -16 | 6 | 267 | 2136 | R Thal |  |
| 5 | -12 | -16 | 6 | 143 | 1144 | L Thal |  |
| 6 | -58 | -24 | 20 | 92 | 736 | L Postcentral gyrus |  |
| 7 | 52 | -18 | 18 | 52 | 416 | R Postcentral gyrus |  |
| 8 | 54 | 10 | 4 | 13 | 104 | R Precentral gyrus |  |
| 9 | 48 | -10 | 16 | 2 | 16 | R middle operculum |  |
| 10 | 20 | -6 | 4 | 1 | 8 | R globus pallidus |  |
| 11 | 56 | -6 | 12 | 1 | 8 | R Precentral gyrus | 43 |
| 12 | 42 | -12 | 14 | 1 | 8 | R Insula | 13 |
| 13 | -46 | -20 | 16 | 1 | 8 | L Insula | 13 |
| 14 | 60 | -26 | 26 | 1 | 8 | R Inferior parietal |  |
| 15 | -4 | 2 | 48 | 1 | 8 | MCC | 24 |
| 1 | 2 | 6 | 44 | 3538 | 28304 | MCC, DMPFC |  |
| 2 | 42 | 10 | 4 | 1939 | 15512 | R Insula, Operculum, VLOFC |  |
| 3 | -14 | -14 | 4 | 1895 | 15160 | L Thal, DS |  |
| 4 | 50 | -14 | 16 | 1780 | 14240 | R Operculum, insula | 43 |
| 5 | 12 | -16 | 2 | 1629 | 13032 | R Thal, DS |  |
| 6 | -58 | -30 | 26 | 1379 | 11032 | L Operculum, L SMG |  |
| 7 | -36 | 8 | -2 | 1339 | 10712 | L Insula |  |
| 8 | 34 | 4 | -10 | 1317 | 10536 | R RTG, R Hipp/parahipp, insula, VLOFC, inferior PFC |  |
| 9 | -42 | -10 | 12 | 1287 | 10296 | L Pre/post central gyrus, insula | 13 |
| 10 | 58 | -32 | 30 | 1245 | 9960 | R SMG | 40 |
| 11 | -32 | -48 | -42 | 1090 | 8720 | Cerebellar Tonsil |  |
| 12 | 26 | -58 | -34 | 1035 | 8280 | Cerebellar culmen |  |
| 13 | 32 | -20 | 62 | 876 | 7008 | R Precentral gyrus |  |
| 14 | 8 | -46 | -24 | 777 | 6216 | Cerebellum Lingual |  |
| 15 | -58 | -10 | 8 | 751 | 6008 | L middle operculum | 22 |
| 16 | 2 | -64 | -36 | 589 | 4712 | Mid Cerebellum |  |
| 17 | -4 | -28 | 30 | 491 | 3928 | PCC | 23 |
| 18 | 48 | 2 | 34 | 475 | 3800 | R Precentral gyrus |  |
| 19 | -56 | 6 | 10 | 454 | 3632 | L Precentral gyrus |  |
| 20 | -20 | -60 | -26 | 366 | 2928 | Cerebellar culmen |  |
| 21 | -28 | -72 | -32 | 205 | 1640 | L Cerebellum/Uvula |  |
| 22 | 58 | -42 | 42 | 178 | 1424 | R Supramarginal gyrus | 40 |
| 23 | 54 | -42 | 16 | 144 | 1152 | R Posterior SMG |  |
| 24 | -56 | -14 | 28 | 139 | 1112 | L Primary Motor | 4 |
| 25 | 6 | -22 | -20 | 108 | 864 | Midbrain/rostral pons |  |

*All results are family-wise whole-brain corrected at p < 0.05

**Supplementary Table 1B: Non-painful aversion-related brain activations in human studies**

**Table 1B** Aversion network in humans

Results of MKDA analysis: Peak voxel activations (upper panel) and peak extended clusters

(lower panel); See Figure 1 for associated activations

Published previously by Hayes & Northoff (2011)

| **Cluster** | **x** | **y** | **z** | **voxels** | **Volume (mm^3^)** | **Brain Region** | **BA** |
| --- | --- | --- | --- | --- | --- | --- | --- |
| 1 | -22 | -2 | -18 | 526 | 4208 | Left amygdala, RTG and hippocampus-parahippocampus |  |
| 2 | 20 | -4 | -14 | 386 | 3088 | Right amygdala, RTG and hippocampus-parahippocampus |  |
| 3 | -36 | 20 | 4 | 27 | 216 | Left anterior insula | 13 |
| 4 | -22 | 18 | -20 | 7 | 56 | Inferior prefrontal gyrus (OFC) | 47 |
| 5 | 40 | 16 | -4 | 4 | 32 | Inferior prefrontal gyrus (OFC) | 47 |
| 6 | -26 | 18 | -16 | 2 | 16 | Inferior frontal gyrus |  |
| 7 | 42 | 16 | -12 | 2 | 16 | Inferior frontal gyrus |  |
| 8 | 10 | -10 | 6 | 2 | 16 | Thalamus |  |
| 9 | -24 | 10 | -28 | 1 | 8 | Rostral temporal gyri | 38 |
| 10 | -10 | 4 | -14 | 1 | 8 | Parahippocampal gyrus | 34 |
| 11 | 44 | 16 | -4 | 1 | 8 | Inferior prefrontal gyrus (OFC) |  |
| 12 | 30 | 10 | 0 | 1 | 8 | Right dorsal striatum (DS) |  |
| 13 | -40 | 16 | 4 | 1 | 8 | Left anterior insula | 13 |
| 14 | 10 | -12 | 10 | 1 | 8 | Thalamus |  |
| 1 | -14 | 2 | -12 | 2276 | 18208 | Left hippocampus-parahippocampal gyrus |  |
| 2 | -34 | 20 | -6 | 1752 | 14016 | Left inferior frontal gyrus |  |
| 3 | -4 | -16 | 0 | 1603 | 12824 | Thalamus |  |
| 4 | 38 | 16 | -4 | 1510 | 12080 | Right inferior prefrontal gyrus (OFC) | 47 |
| 5 | 28 | -6 | -14 | 1486 | 11888 | Right amygdala |  |
| 6 | 14 | 4 | 8 | 1367 | 10936 | Dorsal striatum |  |
| 7 | 32 | 22 | -18 | 1089 | 8712 | Right inferior prefrontal gyrus (OFC) |  |
| 8 | 4 | 24 | 30 | 1084 | 8672 | ACC | 32 |
| 9 | -2 | -10 | 38 | 1000 | 8000 | midACC | 24 |
| 10 | 0 | 52 | 32 | 784 | 6272 | DMPFC |  |
| 11 | -28 | 6 | -28 | 752 | 6016 | Left amygdala/left RTG |  |
| 12 | 10 | -24 | 0 | 578 | 4624 | Thalamus |  |
| 13 | 0 | 8 | 54 | 516 | 4128 | SMA |  |
| 14 | 44 | 34 | 0 | 350 | 2800 | Right inferior frontal gyrus |  |
| 15 | -20 | -44 | -6 | 229 | 1832 | Left parahippocampal gyrus |  |
| 16 | 48 | 0 | -30 | 203 | 1624 | Right middle temporal gyrus |  |
| 17 | 6 | -34 | -16 | 177 | 1416 | Midbrain (area of PAG) |  |

*All results are family-wise whole-brain corrected at p < 0.05

**Supplementary Table 2A**: Painful aversion-related brain activations in animal studies

| **Species** | **Behavioural model** | **Measurement type** | **Specific effect** | **Brain area(s)** | **Reference** |
| --- | --- | --- | --- | --- | --- |
| Wistar rats | Formalin (administered s.c. in lower leg) | c-Fos | Increased activity | PVN of hypothal (mainly in parvo-, over magno-, cellular division); supraoptic n. of hypothal (only regions investigated) | [108] |
| Wistar rats | Formalin- (intraplantar) induced nociception | c-Fos | Increased activity | Lateral septal region (compared only to dorsal and intermediate septal regions) | [109] |
| Sprague-Dawley (SD) rats | Electrical nociceptive forepaw/hindpaw stimulation | fMRI  (nociceptive > baseline); measures of BOLD, CBF, CBV, and CMRO_2_  ^18^F-FDG PET | Increased activity  Decreased activity  Increased activity | Contralateral primary sensory ctx. (Only S1 and striatum (dorsomedial, dorsolateral, ventromedial, ventrolateral) areas investigated.)  Bilateral striatum; particularly ventromedially (BOLD, CBF) and ventrolaterally (CBF).  Contralateral S1. | [174] |
| Wistar rats | Visceral Pain (colorectal balloon) | [^14^C]-iodoantipyrine and autoradiography | female rats showed increased blood flow | Ventral ACC, IL, mid/post Ins, Aud ctx, piriform and temporal association ctx, amyg (central, med, and basomedial n.), thalamus (lat/med geniculate, MDT, VPL, VPM, post., and post. tri-angular n.), NAc, post-vent CP, ext/int GP, RN, pararubral n., prerubral field, SN, ant. pretectal area, endopiriform n., gigantocellular reticular n., hab, INC, LC, IPN, parabrachial area, reticular intermediate n., caudal pontine reticular n., raphe (dorsal, median, paramedian, interpositus nucleus, rostral linear), subbrachial n., SC, IC, and trigeminal, lateral sensory nucleus | [74] |
|  |  |  | female rats showed decreased blood flow | Lat and vent OFC, secondary  somatosensory and dACC, parietal assoc ctx, thal (VLN, VAN, VMN, Ant. n.) |  |
|  |  |  | male rats showed increased blood flow | dACC, ant./post. INS, PL,  S1, S2, M1, M2, V1, V2, A1, A2,  ectorhinal, parietal temporal  assoc ctx, amyg (central n., lat n., anterior dorsal CP |  |
|  |  |  | male rats showed decreased blood flow | VMN, lat CP, RN, pararubral n., prerubral field, SN, PBN n., pons, trigeminal lat sensory n. |  |
| SD rats | Formalin- (intraplantar) induced CPA | c-Fos | Increased expression | ACC, retrosplenial ctx, Ins, parietal ctx area 2, frontal ctx areas 1-3, claustrum, lat septal area, amyg, dorsomedial hyp n., central med. n., PVN, SC, IC, PAG | [156] |
| SD rats | Formalin- (intraplantar) induced CPA  (acquisition)  Acetic acid- (i.p.) induced CPA  (acquisition) | c-Fos | Increased expression | BLA, CeA  CeA, lateral n. amyg | [88] |
| SD rats | Gastric distension | c-Fos | Increased expression | NTS | [110] |
| Lister hooded rats | Electrical nociceptive forepaw stimulation | fMRI  (nociceptive > baseline) | Increased activity  Decreased activity | S1, caudal cerebellum, cuneate n., IC, ant pretectal n., mediodorsal thal n.  PFC, ACC, NAc shell, ventral orbital, IL | [158] |
|  |  | (nociceptive > innocuous stimulation) | Increased activity | S1, S2, cuneate n., IC, pretectal n. |  |
| Wistar rats | Electrical nociceptive stimulation forepaw stimulation  Formalin- (intraplantar) induced nociception | fMRI  (nociceptive > baseline)  c-Fos  c-Fos | Increased activity  Decreased activity  Increased expression  Increased expression | S1, CP  Dorsal horn of cervical spine, CP  Dorsal horn of lumbar spine, CP | [111] |
| Wistar rats | Formalin- (intraplantar) induced nociception | fMRI (nociceptive > baseline) | Increased activity | Cingulate, motor ctx, S1, S2, insula, BNST, V1, V2, CP, Hipp, PAG, SC, Hyp, ventrolateral thalamic group, med thalamus | [79] |
| Wistar rats | Formalin- (intraplantar) induced nociception (rats under ketamine anesthetic) | fMRI (nociceptive > baseline) | Increased activity | Cingulate, motor ctx, S1, S2, insula, NAc, CP, Hipp, ventrolateral thalamic group, med thalamus | [172] |
| Wistar rats | Formalin- (intraplantar) induced nociception | [^18^F]-fluorodeoxyglucose | Increased activity | ACC, M1, S1, S2, insular, visual ctx, CP, Hipp, PAG, Amyg, Thal, and Hyp | [32] |
| Wistar rats | Formalin- (intraplantar) induced nociception (rats under α-chloralose anesthetic) | fMRI (nociceptive > baseline) | Increased activity | ACC, S1, S2, striatum, med thalamus | [173] |
| SD rats | Capsaicin- (intraplantar) induced nociception  Mechanically-induced nociception | fMRI  (nociceptive > innocuous stimulation) | Increased activity | Lateral PAG, thalamus, SC, PBN  Raphe n., IC, deep mesencephalic, pontine reticular, pedunculopontine tegmental n., cuneiform n., lateral lemniscus, superior cerebellar peduncle | [165] |
| SD rats | Formalin- (intraplantar) induced nociception | fMRI (texture analysis; hemispheric control) | Increased activity | Amyg (other regions of interested were investigated) | [182] |
| Wistar rats | Thermal stimulation (on hind paw)  And zymosan-induced (inflammatory) hyperalgesia | fMRI  (nociceptive > innocuous stimulation) | Increased activity  Sensitized increased activity | Cingulate, retrosplenial, S1, S2, M1, insular, piriform, rhinal and parietal association cortices,  pretectal area, basal ganglia, hipp, septal  area, hyp, PAG, thalamus (hab, anterior group, vent. post.lat. n., PVT, lat. post. n., LGN).  Cingulate, retrosplenial, S1, Pretectal/lat. post. thal n., M1, insular, hyp, PAG | [33] |
| SD rats | Formalin- (intraplantar) induced nociception | fMRI  (nociceptive > innocuous stimulation) | Increased activity | Cingulate cortex, somatosensory cortex, amygdala, thalamus, hyp, PAG | [171] |
| SD rats | Capsaicin-induced (intrajoint and intraplantar) nociception | fMRI (nociceptive > baseline) | Increased activity | ACC, S1, M1, frontal ctx | [159,160] |
| SD rats | Formalin- (intraplantar) induced nociception  Electrically- (intraplantar) induced nociception | fMRI  (nociceptive > innocuous stimulation) | Increased activity | ACC, Frontal ctx, insula/piriform ctx  ACC, frontal ctx, S1, S2, M1 | [177] |
| SD rats | Electrically-induced (sciatic nerve) nociception | fMRI  (nociceptive > baseline) | Increased activity | S1, med. thal, cingulate, hyp | [138] |
| Lister hooded rats | Formalin- (intraplantar) induced nociception | c-Fos | Increased activity  Decreased activity | Hipp, rostral ventromedial medulla  Cingulate ctx | [168] |
| SD rats | Gastric-acid-induced pain | c-Fos | Increased activity | NTS | [112] |
|  | Colorectal distention, intrapancreatic bradykinin, intraperitoneal acetic acid | c-Fos | Increased activity | Central lateral nucleus (CL) of the intralaminar thalamus | [113] |
| SD rats | Colon distension | c-Fos | Increased activity | PVN, supraoptic nucleus, accessory neurosecretory n. of hyp, NTS, ventrolateral medulla, LC, Barrington n. | [179] |
| Slc-ddY mice | Formalin- (intraplantar) induced nociception | c-Fos | Increased activity | PFC, BLA, CeA | [114] |
| Wistar rats | Electrically- (hindpaw) induced nociception | c-Fos | Increased activity | Hyp n.: anterior n., PVN, VM n., DM n., LH, posterior Hyp. | [115] |
| SD rats | Electrically- (hind toe) induced nociception | c-Fos | Increased activity | Anterior BNST | [116] |
| Wistar rats | Electrically- (knee) induced nociception | c-Fos | Increased activity | Hyp: anterior n., posterior area | [117] |
| SD rats | Intra-stomach 2% formalin | c-Fos | Increased activity | PBN (and subnuclei) | [118] |
| SD rats | Colon distension | c-Fos | Increased activity | PVN and supraoptic n. of hyp., LC-Barrington's n. complex, area postrema, NTS, cingulate ctx, posterior PVN of thal., PAG, ventrolateral medulla | [163] |
| Lister hooded rats | Pinch- and electrically- (hindpaw) induced nociception | c-Fos | Increased activity | SC | [119] |
| Wistar rats (divided into high and low sensitivity to pain) | Electrically-induced nociception | c-Fos | Increased activity | Lat hab, PAG, BLA, CP, cingulate, paraventricular ventricular n., parietal ctx  High > low sensitivity: Lateral hab, BLA | [155] |
| SD rats | IP injection of acetic acid (somato-visceral) pain | c-Fos | Increased activity | cerebral cortex, lateral septum, parastriatal n, septohypothalamic n, amyg  (med, anterior cortical and basomedial nuclei), thalamus (paraventricular and central med nuclei), hyp (paraventricular, supraoptic, dorsomedial, retrochiasmatic, arcuate and lateral nuclei), PAG, laterodorsal tegmental n, LC, lateral PBN, DRN, MRN, med vestibular n, dorsal cochlear n, lateral reticular n, NTS, raphe pallidus, cuneate n and the ventrolateral medulla | [175] |

**Supplementary Table 2B: Aversion-, non-painful, related brain activations in animal studies**

| Species | Behavioural model | Measurement type | Specific effect | Brain area(s) | Reference |
| --- | --- | --- | --- | --- | --- |
| Long-Evans rats | Ferret odor exposure | c-Fos | Increased expression | Posterior medial n. of amyg (only amyg was investigated) | [107] |
| Wistar rats | Cat odor exposure | c-Fos | Increased expression | Posterior basomedial n., anterior cortical n., lateral n., medial n. (only subdivisions of amygdala were investigated) | [162] |
| Wistar rats | Foot-shock-induced freezing | c-Fos | Increased expression | Cg, PL, OFC, RAIC, midline and intralaminar thalamic nuclei (CM, IMD, Rh, Re), LA, LH, Hab, NAc shell, PeF, rostromedial tegmental n., vlPAG | [146] |
| Mice | LiCl-induced (130 mg/kg; i.p.) CPA(acquisition/exposure) | c-Fos | Increased expression | Cingulate, paraventricular hypothalamic n. (PVN) (significant for both CPA expression and cocaine-induced CPP) | [151] |
|  |  |  | Decreased expression | Dentate gyrus (significant for both CPA expression and cocaine-induced CPP) |  |
|  | LiCl-induced (130 mg/kg; i.p.) CPA(expression) |  | Increased expression(CS+ > CS-) | Cingulate, paraventricular hypothalamic n. (PVN) (significant for both CPA expression and cocaine-induced CPP); paraventricular thalamic n.; PAG |  |
| Wistar rats | Intra-PAG semicarbazide-induced (5µg; GABA synthesis inhibitor) CPA | c-Fos | Increased expression(CS+ > CS-) | dmPAG, BLA, laterodorsal n. of the thal. | [183] |
| Wistar rats | CTA with strawberry flavoured water paired with intragastric hypertonic (5%) NaCl injection | c-Fos | Increased expression when NaCl followed CS+ exposure (though not with a 30 min delay) | Intermediate n. of the solitary tract (iNST; only nucleus investigated) | [104] |
| Sprague-Dawley (SD) rats | Taste-potentiated odor aversion (TPOA), simultaneous CTA and conditioned odor aversion with saccharin combined with LiCl (0.2M; i.p.) | c-Fos | Olfactory or taste cue: increased expression | Anterior paleocortex, posterior paleocortex, entorhinal ctx, hippocampus (CA1/3), BLA, medial n. amyg, OFC, dysgranular insula | [141] |
| Wistar rats | CTA following arsenic administration (20 mg/kg) | c-Fos | Increased expression | Central n. amyg., BNST, NST | [147] |
| SD rats | CTA by LiCl (0.4M; i.p.) | c-Fos | Increased expression | Central n. amyg, BLA, PBN, BNST, gustatory thalamus | [176] |
| SD rats | CTA by LiCl (0.15M; i.p.) | c-Fos | Increased expression | Central n. amyg, BLA, PBN, NST, insular (gustatory) ctx | [137] |
| Wistar rats | CTA by LiCl (127 mg/kg; i.p.; acquisition) | c-Fos | Increased expressionDecreased expression | Lateral n., central n., basolateral n. amyg.NAc core | [144] |
| Wistar rats | CTA by LiCl (0.15M; i.p.; expression) | c-Fos | Increased expression(CS+ > CS-) | Insula, NAc shell | [181] |
|  | CTA by LiCl(acquisition) |  | Increased expression | Central n. amyg., BNST |  |
| Wistar rats | CTA by LiCl (0.2M; i.p.)(expression) | c-Fos, EGR1EGR1 (alone) | Increased expression | Medial portion of the central n. amyg., BLA, NAc shell and core, interstitial n. of the posterior limb of the anterior commissure | [140] |
|  | TPOA with LiCl |  | Increased expression | BLA, insula, hippoCentral n. amyg, entorhinal ctx |  |
| Long-Evans rats | CTA with LiCl (0.15M; i.p.)Conditioned intra-oral aversion | c-Fos | Increased expression | Central n. amyg, BLA, insula, NSTCentral n. amyg | [180] |
| Long-Evans rats | CTA with LiCl (0.15M; i.p.) and novel stimuli | c-Fos | Increased expression | Central n. amyg, BLA, insula, iNST, PBN, | [152] |
| SD rats | CTA with LiCl(81 mg/kg; i.p.; acquisition)CTA with LiCl (expression) | c-Fos | Increased expression | Central n. amyg, BLA, iNSTBLA, iNST | [164] |
| Wistar rats | Freezing and escape behaviour elicited by electrical stimulation of the dorsolateral PAG | c-Fos | Increased expression induced by freeze-inducing stimulationIncreased expression induced by escape-inducing stimulation | Dorsomedial PAG, dorsal premammilary n.Dorsomedial PAG, dorsolateral PAG, ventromedial hypothal, dorsal premammilary n., cuneiform n. | [178] |
| Long-Evans rats | CTA with LiCl (0.15M; i.p.; one conditioning trial)CTA with LiCl (3 conditioning trials) | c-Fos | Increased expression | iNSTiNST, PBN, central n. amyg | [166] |
| Wistar rats | LiCl-induced (0.15M; i.p.) CTA(retrieval of aversive memory following CS+ exposure) | fMRI (manganese-enhanced) | Increased activity | Gustatory (insula) cortex, NAc core and shell, VP, LH, Central n. amyg, BLA | [78] |
| Wistar rats | LiCl-induced (0.15M; i.p.) CTAFoot-shock-induced avoidance | c-Fos | Increased expressionIncreased expression | Anterior nuclei of the thalamus: no changeMidline and intralaminar thalamic complex: PVTAnterior nuclei of the thalamus: Anterodorsal n.Midline and intralaminar thalamic complex: PVT | [91] |
| SD rats | CTA with LiCl (0.15M; i.p.); investigation of amyg only using laser capture and RT-PCR | c-Fos, Fra-2 | Increased expression | Central n. amyg (c-Fos, Fra-2), BLA (Fra-2) | [106] |
| SD rats | Exposure to predatory fox odour compared to control, butyric acid | c-Fos | Increased expression | olfactory bulb, lateral septal n, septohypothalamic n, anteromedial and oval nuclei of the BNST, CeA, the anteroventral, anterodorsal, and medial preoptic nuclei, the anterior, dorsomedial, lateral, supramammillary, dorsal premammillary and paraventricular hypothalamic nuclei, the external lateral PBN, LC, NST | [41] |
| Wistar rats | Exposure to predatory cat odour compared to control | c-Fos | Increased expression | posteroventral medial amygdaloid nucleus, the premamillary nucleus (dorsal part), ventromedial hypothalamic nucleus (dorsomedial part), dorsomedial hypothalamic nucleus, periaqueductal gray (dorsomedial, dorsolateral and ventrolateral parts) and the cuneiform nucleus | [142] |
| Wistar rats | Odour-conditioned (to footshock) | c-Fos | Increased activity | olfactory bulb, infralimbic cortex, OFC, perirhinal-entorhinal ctx, BLA | [145] |
| Wistar-Kyoto rats | Cold chamber (4°C/3 hrs) | c-Fos | Increased activity | rostral thal, zona incerta, midline thalamic, hypothal dorsomedial, supramamillary and lateral PBN, PVN hypothal, arcuate, CeA, NST | [136] |
| SD rats | Hypercarbic chamber | c-Fos | Increased activity | Hypothalamus (DMH, PeF, PVN, PMd), PAG, rostroventrolateral medulla, lateral paragigantocelluar n. | [150] |
| Wistar rats | Exposure to footshock-paired chamber | c-Fos | Increased activity | PL/IL | [157] |
| SD rats | Intragastrically administered bitter tasting-receptor ligands (10mM) | c-Fos | Increased activity | Area postrema, NST, PBN, PVN hypothal, CeA | [148] |
| Wistar rats | Social defeat | c-Fos | Increased activity | Arcuate n, ventromedial n of the hypothal, and medial amygdala | [143] |
| SD rats | Cue-associated footshock and footshock alone | c-Fos | Increased activity | Core of the rostromedial tegmental n (projecting to VTA), SNR | [149] |
| Mice | LiCl-induced (0.14M; i.p.) CTA | c-Fos and Zif268/Egr1 | Increased activity | Amyg (Zif268 only) | [105] |
| Wistar rats | Playback of 22 kHz aversive vocalizations | c-Fos | Increased activity | perirhinal cortex, amygdalar nuclei, PAG | [170] |
| Wistar rats | Conditioned freezing to footshock-paired compartment | c-Fos | Increased activity | M2 ctx, PVN, BLA, CeA, MeA, CA1, DG, DRN | [154] |
| Mice | Tone-conditioned footshock-induced aversionFootshock-induced aversion | c-Fos | Increased activity | Ventrolateral septum, dorsolateral septumVentrolateral septum, medial septum | [90] |
| Wistar rats | Elevated plus maze exposure | c-Fos | Increased activity | PL, IL, BLA, CeA, ACC | [135] |
| SD rats | Predator (fox) scent | MnCl_2_-enhanced fMRI (aversive scent > neutral scent) | Increased act. ipsil.Decreased act. Ipsil.Increased act. contra.Decreased act. contra. | Thal, hypothal, amygPFCNonePFC | [139] |
| Wistar rats | Social defeat | c-Fos | Increased activity | Hippocampus (CA1, CA2, CA3, DG) | [102] |
| Macaques | Threatening faces | fMRI (threat > pleasant) | Increased activity | BLA, superior temporal sulcus, inferotemporal cortex | [103] |
| SD rats | Visual exposure to predator (ferret) | c-Fos | Increased activity | MeA, CeA, BLA, Lat habenula, PVN of the thal, hypothal (lat n, dorsal premammillary n) | [169] |
| Wistar rats | Open field exposure | c-Fos | Increased activity | MeA, CeA | [101] |
| Wistar rats (bred for high vs. low anxiety) | Airjet (compressed air) | c-Fos | Increased activity (both high and low anxiety)Increased activity (high > low anxiety) | mPFC, ACC, caudate putamen, NAc, lat septum, PVN of the thal, hypothal, amyg, PAG, VTA, DR, latPBN, LCAnterior hypothal, med preoptic area, dorsolateral PAG, LC | [128] |
| Wistar rats | Tone-conditioned footshock; avoidance of footshock | c-Fos, P-ERK | Increased activity | Lat dorsal amyg (ventral portion) | [100] |
| SD rats | Footshock exposure | c-Fos | Increased activity | Amyg, thal, hypothal | [167] |
| Wistar rats | IC electrical stimulation causing:FreezingEscape | c-Fos | Increased activityIncreased activity | Frontal ctx, BLA, dorsal hippo, entorhinal, CeAFrontal ctx, BLA, dorsal hippo, dPAG, cuneiform n, IC | [153] |
| SD rats | Footshock exposure | NGFI-B | Increased activity | Lat dorsal amyg, hippo (CA1), neocortex | [161] |
